# Assessment of Compatibility between Various Intraoral Scanners and 3D Printers through an Accuracy Analysis of 3D Printed Models

**DOI:** 10.3390/ma13194419

**Published:** 2020-10-04

**Authors:** Chang-Hee Im, Ji-Man Park, Jang-Hyun Kim, You-Jung Kang, Jee-Hwan Kim

**Affiliations:** Department of Prosthodontics, Yonsei University College of Dentistry, 50-1 Yonsei-ro, Seodaemun-Gu, Seoul 03722, Korea; zol_bayar_nass@yahoo.com (C.-H.I.); JIMARN@yuhs.ac (J.-M.P.); 881004kjh@yuhs.ac (J.-H.K.); kyj1219@yuhs.ac (Y.-J.K.)

**Keywords:** dental computer aided manufacturing (CAD-CAM), intraoral scanner, 3-dimensional printer, quadrant model, trueness

## Abstract

To assess the accuracy of various intraoral scanners (IOSs) and to investigate the existence of mutual compatibility that affects the accuracy between IOS and 3-dimensional (3D) printing using a scan quadrant model. For clinical implication, crown preparations and cavity design according to prosthetic diagnosis and treatment considerations must be acquired by a digital scanner. The selected typodont model was scanned using a reference scanner, from which reference (Ref) standard tessellation language (STL) data were created. Data obtained by scanning the typodont model with IOSs based on three different technologies were divided into three groups (CS3600, i500, and Trios3). Scanned data from the groups were divided into sub-groups of digital light processing (DLP), fused deposition modeling (FDM), and stereolithography apparatus (SLA), based on which 3D printed models (3DP) were fabricated. The 3DP dental models were scanned to obtain a total of 90 3DP STL datasets. The best-fit algorithm of 3D analysis software was used for teeth and arch measurements, while trueness was analyzed by calculating the average deviation among measured values based on superimposition of Ref and IOS and 3DP data. The differences between Ref and IOS (Ref-IOS), Ref and 3DP (Ref-IOS/3DP), and IOS and 3DP data (IOS-3DP) were compared and analyzed, while accuracy within each of the three main groups was assessed. For statistical analysis, the Kruskal–Wallis, Mann–Whitney *U*, and repeated measures ANOVA test were used (*p* < 0.05). The major finding is that the mutual relationships between IOSs and 3D printers vary depending on the combination. However, i500 intraoral scanner and DLP 3D printer was the combination that showed the best trueness value.

## 1. Introduction

In the world of data and digital technology, dental treatment has benefitted significantly from rapid advances in computer-aided design and computer aided manufacturing (CAD/CAM) technology, making dental clinical practice much more convenient [1,2,3,4,5]. With the introduction of various products based on new technologies, difficult and complex procedures using aligners, inlays, onlays, and coverage crowns became more affordable, efficient, and simpler. Moreover, it became easier to obtain direct data, which could then be acquired and transmitted by digital platforms [1,2,4,5,6,7,8,9,10,11]. By using such new technologies, we are able to improve diagnostic accuracy and treat patients in a more effective and comfortable manner, while also being able to provide a safer, faster, and more comfortable experience through greater availability of CAD/CAM technology [2,5,7,12,13,14]. Currently, CAD/CAM technology is being applied more quickly in dentistry than in other industries. Owing to technical advances in artificial intelligence and big data for impression taking with three-dimensional (3D) scanners and CAD processes, dentists and dental technicians can easily connect with each other anywhere in the world [2,4,5,15].

The increased availability of intraoral scanner (IOS) technology in the dental field is expanding the applicable range of 3D printed dental models for fabrication of customized products. As a result, IOS has been used often according to digital workflow in the past 7 years, with increasing use for fabricating the final restoration by printing the scanned data obtained from dental restorations with a 3D printer. It has become possible to actively analyze data, plan restorations in virtual space, directly fabricate anatomical models with a 3D printer, and materialize dental restorations, including occlusal devices, aligners, retainers, bridges, veneers, partial denture framework, dental implants, and computer-guided implant templates. Meanwhile, the successful use of 3D printers is being applied to patients and increasing in clinical practice [4,5,15,16,17,18,19]. However, assessment of compatibility with respect to the matching process between two types of equipment with different functional principles remains somewhat unclear; 3D printers toughen resin layers with a digital optical projector or a laser beam and IOSs are a part of limited optical equipment with a depth of field that varies depending on the system. Accordingly, comparative studies on this topic are needed [1,2,3,4,9,17,19].

Differences in the accuracy of IOS could lead to differences in restoration fabrication accuracy and fit when installed in the mouth. Minute errors during digital impression taking could cause biological and mechanical complications due to prosthesis misfit; the clinically acceptable marginal discrepancy (width) must be less than 120 μm [2,20]. Therefore, to fabricate a model that is accurate enough to result in restoration with excellent fit, the overlapping area of image acquisition must not be distorted during intraoral scanning and standard tessellation language (STL) format reconstruction [2,21]. When digital impression taking using IOS and traditional impression taking are compared, the digital method, which uses IOS to capture optical images as well as various software programs that are being upgraded, could save time and cost, while enabling the fabrication of dental restorations, including 3D models. As a result, there are various modifications and versions of digital products in today’s market, and software technology has become important, as the equipment can save time and accelerate the workflow in modern dental clinics [4,5,8,14,22,23]. For the features of each IOS method, especially the precision and accuracy of a product, the fact that there are favorable functions preferred over others must be considered [24,25]. IOSs are very popular owing to various advantages, and in particular, the latest digital impressions allow for complete-arch scanning at clinically allowable level, and many studies have been conducted to assess the accuracy between various IOSs [2,14,26,27,28], Renne et al. reported an in vitro assessment of the accuracy of seven types of digital complete-arch scanners and stated that it is necessary to have a good understanding of the fact that each scanner has its own unique advantages and disadvantages [26]. Therefore, varying levels of accuracy may appear depending on the equipment characteristics of each IOS; it is necessary to identify issues that should be considered for clinical application.

Digital techniques involved in IOS data processing and designing and printing the data with a 3D printer have recently drawn much interest [17,29,30]. Various types of 3D structures could be fabricated using STL data obtained from IOS, and various dental devices could be fabricated with 3D printers. However, there is a lack of studies on IOS based on which principle and what type of 3D printers have high compatibility and are superior [9,10,18]. Therefore, it is necessary to study whether accurate results could be obtained through print tests using data obtained from IOS printed using various types of 3D printers. Digital manufacturing can be divided into two types: additive manufacturing (AM) and subtractive manufacturing (SM). AM involves adding layer upon layer until the final model is produced. In contrast, SM, which is also referred to as milling, involves cutting a block to the desired shape. Compared to SM, such as milling hard ceramic, AM, which lacks resolution and accuracy, can be used to produce complex structures without wasting material unlike SM where material is discarded after being cut [4,5,18,19,31].

A study by Nestler et al. assessed the accuracy of complete-arch casts using various 3D printers, including fused deposition modeling (FDM), stereolithography apparatus (SLA), and digital light processing (DLP) technologies, and identified that the deviation between printers with respect to trueness was 12 to 240 μm. This study concluded that the DLP 3D printer showed the highest overall accuracy. Therefore, because technologies related to 3D printers have varying levels of trueness, this should be considered when the use of technology is assessed in dentistry and prosthodontics [9,32].

The present study aimed to find the optimal combination of a scanner and printer with different attributes to obtain the best possible results that affect the accuracy between digital impression taking and 3D printing through IOS [1,2,3] according to the dentition of quadrant model geometry that needs planned prosthodontic treatment. Various crown preparations and cavity designs according to prosthetic diagnosis and treatment considerations must be acquired by a digital scanner through superimposition of the product. Because there are almost no studies on the compatibility between 3D printed products, clinical assessment must be performed. Keeping this in mind, the objective of the present study was to assess how much error there is as compared to the original model to determine how much influence there is on accuracy according to the compatibility of 3D products after IOS and 3D printer printing processes. For this, quadrant models with various crown preparations and cavity designs were scanned with three different IOSs, after which, scanned data were converted to a 3D resin model using three different 3D printers. The deviations for each part were compared between the reference scan data and the 3D printing data, and between the reference scan data and IOS data. The interoperability and effects of IOS and 3D printers were also evaluated. The null hypothesis was that there would be no differences in the accuracy between the trueness after the 3D printing process alone and that of IOS scanning and 3D printing.

## 2. Materials and Methods

### 2.1. Study Quadrant Phantom Model Design

**Figure 1:**
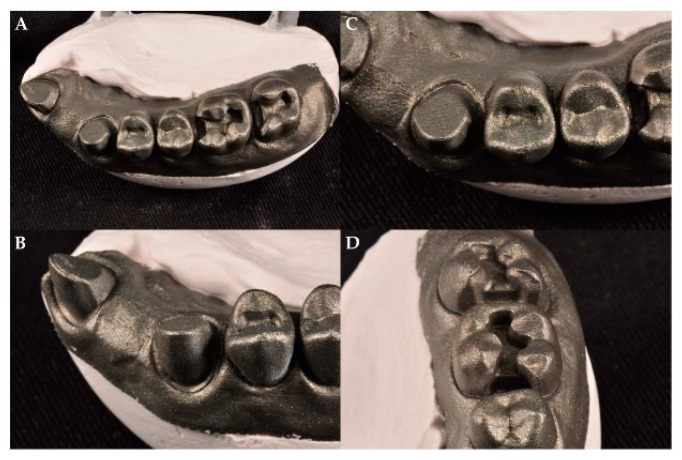
(**A**) 3-dimensional (3D) printed right maxillary quadrant Co-Cr typodont model, (**B**) Complete coverage crown preparation on maxillary canine (MxC#13), (**C**) Occlusal inlay preparation on maxillary first premolar (MxFPM#14), (**D**) Mesio-occlusal inlay preparation on maxillary first molar (MxFM#16).

The present study used a maxillary complete-arch artificial dental model (A50H-Set; J. Morita Europe GmbH, Dietzenbach, Germany) fabricated with epoxy resin that was prepared for various inlays and complete coverage crowns. The artificial tooth model was scanned using a digital impression industrial precision scanner (stereoSCAN neo; AICON 3D Systems GmbH, Braunschweig, Germany). Scanned STL data were used, and the direct metal laser sintering technique was used to fabricate a maxillary right quadrant phantom model with cobalt-chromium (Co-Cr) material using a 3D printer (Eosint M270; EOS GmbH, Krailling, Germany). The types and locations of various inlays and complete coverage crowns prepared with the quadrant model were as follows: a complete coverage crown preparation on a maxillary central incisor, a mesio-occlusal inlay preparation on a maxillary second molar, a mesio-occlusal inlay preparation on a maxillary first molar (MxFM#16), an occlusal inlay preparation on a maxillary first premolar (MxFPM#14), and a complete coverage crown preparation on a maxillary canine (MxC#13) (Figure 1). Metal typodont is used to prevent from distortion or volume change due to the experimental conditions such as temperature and humidity. Heat application on metal typodont made its surface like it is sandblasted, resulting in no light distortion. So, there is no need to use powder on metal typodont [33]. During the experiment, the quadrant model was not altered by the addition or removal of teeth or the application of external force. All experimental actions were performed under experimental conditions, with a temperature of 23 ± 1 °C and a relative humidity of 50 ± 5%.

### 2.2. Digital Scanning Process

To obtain reference (Ref) data, the typodont model was scanned once using a T500, blue light-emitting diode high-accuracy tabletop scanner (Identica T500; Medit Inc., Seoul, Korea), which was used as the reference scanner. Scanned data were saved as an STL reference file. For the next process, three different IOSs were used to scan the phantom model to acquire STL data, and data were divided into three groups. Table 1 shows the scanners that were used: CS3600 (Carestream, Rochester, NY, USA); i500 (Medit, Inc., Seoul, Korea); and Trios3 (3Shape A/S, Copenhagen, Denmark). For the scan process, 10 scans were completed by each scanner and a total of 30 scanned datasets named from data obtained from IOS (*n* = 10 for each group) were generated. The scanning procedure was conducted according to the manufacturers’ instructions by one trained, experienced operator. In the next process, STL data from the IOS group were used, and 3D models were fabricated with the next AM technique connected to three different 3D printers. Each 3D printed model has a unique printing method. Table 2 shows the types of 3D printers used: DLP (D2; Veltz3D, Incheon, Korea); FDM (Creator Pro; Flashforge, Zhejiang, China), and SLA (Form2; Formlabs, Somerville, MA, USA). During the experiment, the 3D models were not altered by the addition or removal of teeth or application of external force. Each completed 3D model was scanned using the colLab Scan software (2017 v.2.0.0.4; Medit Inc., Seoul, Korea) of the T500 reference scanner to obtain 3D printed model data (3DP) (*n* = 30 for each printer group), and as a result, a total of 90 sets of STL model data named according to the printer were generated. The 3D printer model data were divided into sub-groups.

### 2.3. Three-Dimensional Analysis

**Figure 2:**
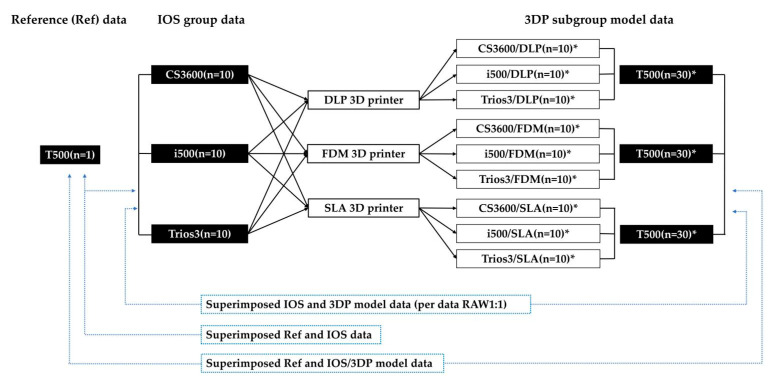
Three main groups (Ref-IOS, Ref-IOS/3D printed models (3DP), and IOS-3DP data) of the superimposed data read workflow diagram. (* Each printed model was scanned with a tabletop scanner to obtain standard tessellation language (STL) data).

**Figure 3:**
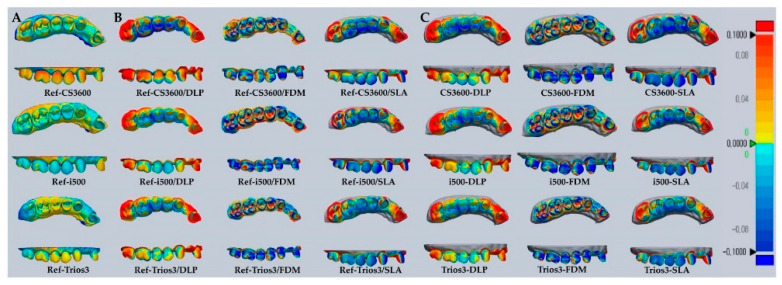
Representative deviations of superimposition using a surface matching software on the 3D digital model. Range of deviation was color coded from −100 (blue) to +100 µm (red). (**A**) superimposition between Ref-IOS data. (**B**) superimposition between Ref-IOS/3DP data. (**C**) superimposition between IOS-3DP data.

There was superimposition of 121 STL data sets by 3D software analysis (Geomagic Verify X v.4.1.0.0; 3D Systems) and a total of 210 measured digitized data sets were generated by Geomagic 3D analysis software. For the trueness evaluation, alignment between the reference data and scanned experimental data was conducted by the best-fit algorithm. The median trueness of Ref-IOS data, Ref-IOS/3DP data, and IOS-3DP superimposed data were calculated. The overall data evaluation workflow is presented in Figure 2. To evaluate the magnitude and distribution of the digitized quadrant-arch model set acquired by the superimposition of Ref-IOS, Ref-IOS/3DP, and IOS-3DP data, color-coded map diagrams were drawn in the inspection software (Figure 3).

### 2.4. 3D Printing Process

To obtain perfect quality, printable model data, function from the Model Creator module of Exocad software (Dental CAD software v.6136; Exocad GmbH, Darmstadt, Germany) was used. The quality of the entire area of STL data scanned using this software was checked, and unscanned empty areas were filled and the marginal areas of scanned data were organized to prepare STL data. Organized STL data were used to print with three different 3D printers to produce the 3D printed models. The build orientation of products printed by a DLP printer was set to 0° and the layer thickness of the prosthesis was 100 μm. The dental models were printed using resin (DM-1, 3D system) material. The occlusal surface of the printed model was connected vertically to the support structure to achieve 0° build orientation. Each printed model was washed for 20 min using 95% isopropyl alcohol (IPA) (wash produced by the Form manufacturer) before drying. Subsequently, an ultraviolet ray curable unit (60°, MP100; Hephzibah, Incheon, Korea) was used for 30 min to cure and harden the model to obtain 30 completed 3D DLP models. The build orientation of products printed by a FDM printer was set to 0° and the layer thickness of the prosthesis was 100 μm. The dental models were printed using a PLA/PHA flexible filament (standard white d1.75 mm) material. Subsequently, an ultraviolet ray curable unit (60°, P100; Hephzibah, Incheon, South Korea) was used for 30 min to cure and harden the model to obtain 30 completed 3D FDM models. The build orientation of products printed by an SLA printer was set to 0° and the layer thickness of the prosthesis was 100 μm. The dental models were printed using grey resin (V4, Photopolymer resin) material. Each printed model was washed for 20 min using 95% IPA with a form washing machine from the manufacturer before drying. Subsequently, an ultraviolet ray curable unit (MP100; Hephzibah, Incheon, Korea 60°) was used for 30 min to cure and harden the model to obtain 30 completed 3D SLA models. The 3D printers were prepared using preparation instructions given by each manufacturer, and a total of 90 printed models were produced.

### 2.5. Comparative Photo Analysis Through the Experimental Process

**Figure 4:**
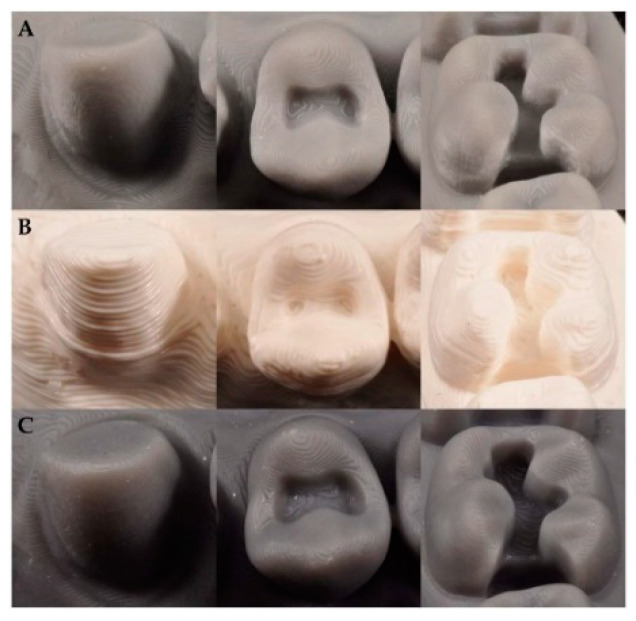
Surface characteristics of three different types of ((**A**) digital light processing (DLP), (**B**) fused deposition modeling (FDM), and (**C**) stereolithography apparatus (SLA)) 3D printed models.

**Figure 5:**
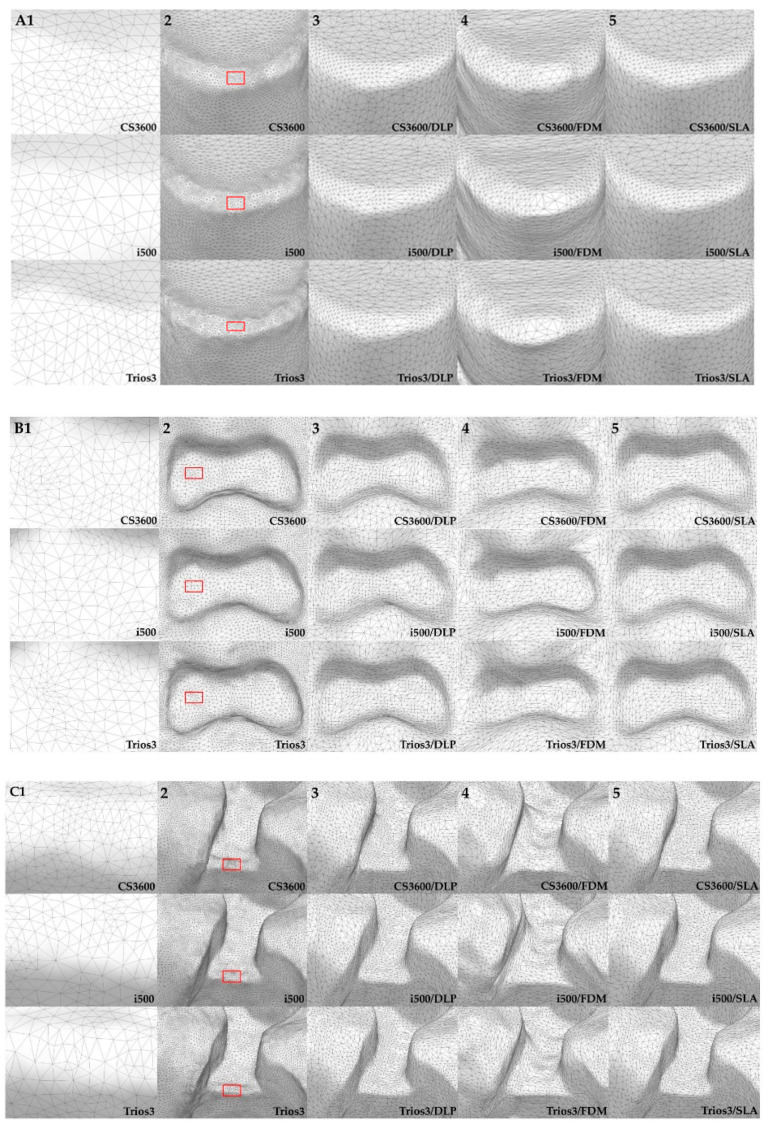
(**A**) The teeth #13 (MxC), (**B**) #14 (MxFPM), and (**C**) #16 (MxFM). The numbers of per unit area compared in terms of the density and geometry of the triangle polygons for qualitative evaluation of each intraoral scanners (IOSs) and 3DP STL data.

**Figure 6:**
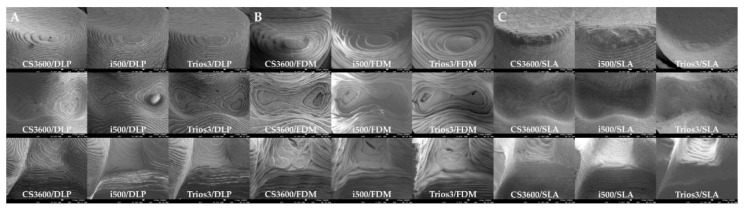
(**A**) DLP, (**B**) FDM, and (**C**) SLA each of the 3D printed models-SEM images at the three type surface characteristics of the models in the layer thickness density and shape by layer technique for qualitative evaluation (original magnification 30×).

Prepared 3D models were photographed using a zoom lens of a Nikon AF-S Micro-NIKKOR 105-mm f/2.8G IF-ED lens camera. The three types of teeth that were selected #16 (MxFM), #14 (MxFPM), and #13 (MxC) were visualized for edge sharpness and the surface smoothness specific margin (Figure 4). Rapidform 2006 software (INUS Technology, Seoul, Korea) was used, and according to the tooth shape (#16, #14, and #13), IOS data and 3DP data were compared within isolated areas for visualization of the geometric assessment of the triangle polygon density and shape (Figure 5). Microstructures and qualities of 3D printed models were compared and visualized with field emission scanning electron microscope (FE-SEM) images (Figure 6).

### 2.6. Statistical Analysis

Statistical analyses were conducted using SPSS (IBM Statistics, v.25.0; IBM Corp, Armonk, NY, USA), and all analyses were performed with a significance level of α = 0.05. The data was analyzed not to follow the normal distribution after the Kolmogorov–Smirnov test of three major groups, nonparametric analysis was conducted. The median trueness values of the three major groups were analyzed using the Kruskal–Wallis test, after which, the Mann–Whitney *U* test was used for paired comparisons between data from each group. Interactions between scanners were assessed using post-hoc testing with a Bonferroni adjustment (*p* < 0.05). To identify the differences between IOS-3DP and Ref-IOS/3DP deviations, a repeated measures analysis of variance (ANOVA) was performed, while the results from both IOS and 3D printing were compared and tested against analysis results from 3D printing. For mean estimated values, the Bonferroni test was used for pairwise comparisons (*p* < 0.05).

## 3. Results

Comparisons between IOSs and the median trueness values of IOS-3DP and Ref-IOS/3DP are shown in Table 3. Table 4 shows the results of analyzing the relationship between IOSs and 3D printers by identifying the differences between deviations of IOS-3DP and Ref-IOS/3DP using raw data from IOS-3DP and Ref-IOS/3DP.

### 3.1. Trueness of Only the IOS Process

For accuracy analysis between IOSs, reference data of the typodont model were overlapped with IOS data and trueness values were compared. The results showed that the deviation between Ref-i500 (23.5 μm) was significantly lower than the deviation between Ref-CS3600 (30.2 μm) (*p* < 0.05). There were no significant differences in the deviation values between Ref-Trios3 and Ref-CS3600, and between Ref-Trios3 and Ref-i500 (*p* < 0.05, Table 3).

### 3.2. Trueness of IOS and 3D Printing Processes

To assess the changes in accuracy during the process of undergoing IOS and 3D printing processes, reference data of the typodont model (Ref) and data from scanning the 3D printed model in each group (3DP) were compared (Table 3). In the DLP printer group, the median trueness of Ref-CS3600/DLP (59.5 μm) was significantly higher than that of Ref-i500/DLP (43.2 μm) and Ref-Trios3/DLP (44.8 μm) (*p* < 0.05). There was no significant difference in the trueness values between Ref-i500/DLP and Ref-Trios3/DLP (*p* > 0.05). In the FDM printer group, the median trueness of Ref-CS3600/FDM (64.3 μm) was significantly lower than those of Ref-i500/FDM (81.9 μm) and Ref-Trios3/FDM (78.8 μm) (*p* < 0.05). There was no significant difference in the trueness values between Ref-i500/FDM and Ref-Trios3/FDM (*p* > 0.05). In the SLA printer group, the median trueness of Ref-i500/SLA (65.5 μm) was significantly higher than that of Ref-Trios3/SLA (56.6 μm) (*p* < 0.05). There was no significant difference in the trueness values between Ref-CS3600/SLA, Ref-i500/SLA, Ref-CS3600/SLA, and Ref-Trios3-SLA (*p* > 0.05). In the comparison of Ref-IOS/3DP that underwent both IOS and 3D printing, the combination that showed the best trueness was the i500 IOS plus DLP printer.

### 3.3. Trueness of the 3D Printing Process

For accuracy analysis of only the 3D printer, data from scanning the typodont model with each IOS were compared with data from scanning the product printed with each 3D printer (Table 3). In the DLP group, the median trueness of CS3600-DLP (51.8 μm) was significantly higher than that of i500-DLP (46.2 μm) (*p* < 0.05). There were no significant differences between Trios3-DLP, i500-DLP, and CS3600-DLP (*p* > 0.05). In the FDM group, the median trueness of CS3600-FDM (73.3 μm) was significantly lower than those of i500-FDM (77.6 μm) and Trios3-FDM (78.8 μm) (*p* < 0.05). There was no significant difference in the trueness values between i500-FDM and Trios3-FDM (*p* > 0.05). In the SLA group, there were no significant differences in the trueness values between CS3600-SLA (61.5 μm), i500-SLA (60.2 μm), and Trios3-SLA (61.2 μm) (*p* > 0.05). IOS data were directly fed to each 3D printer, and thus, assessment considering only the 3D printing process showed the best results when i500 data were printed with a DLP printer.

### 3.4. Analysis of Differences between IOS-3DP and Ref-IOS/3DP Deviations

Table 4 shows the results of a repeated measures ANOVA performed to analyze the relationships between IOSs and 3D printers using the differences between IOS-3DP and Ref-IOS/3DP deviations. When printing with an DLP printer, all IOS groups showed differences in the comparisons of only printing and comparisons of both IOS and printing (*p* < 0.05). When printing with an FDM printer, Trios3 did not show a statistical difference (*p* > 0.05). When printing with an SLA printer, all IOS groups showed differences (*p* < 0.05).

### 3.5. Comparative Photo Result

Figure 3 shows the trueness deviation pattern representative of each data set maxillary quadrant digital model in the present study. Color-coded deviation maps were used to mark digitized data capture in occlusal and buccal directions, and comparisons between groups were visualized. The Ref-IOS group was compared against Ref-IOS/3DP and IOS-3DP groups, showing moderate deviation with a yellow/light blue color. On the other hand, Ref-IOS/3DP and IOS-3DP groups showed large deviations with a red/blue color. From an occlusal view, the middle section corresponding to the premolar in the quadrant arch showed blue-colored contraction and both ends showed red-colored expansion. Particularly in comparisons of Ref-IOS/DLP, IOS-DLP, Ref-IOS/SLA, and IOS-SLA, the SLA groups show large deviations with darker colors. From a buccal view, both the FDM and SLA groups showed large deviations. However, Ref-IOS/FDM and IOS-FDM showed a red-colored expanded deviation pattern in the intra-coronal cavities (dimples), but mostly blue-colored contracted deviation patterns in all other areas.

In the present study, a digital single-lens reflex camera was used to photograph teeth #13 (MxC/75°), #14 (MxFPM/90°), and #16 (MxFM/45°) at three different angles (75°, 90° and 45°) from three models that were 3D printed using three different methods (Figure 4). In the comparison of printed layer morphology by printer group, printing with a DLP 3D printer showed a somewhat jagged and thin smooth layer margin (Figure 4A). When printed with an FDM 3D printer, the margin did not appear prominently, and an insufficient thick layer appeared (Figure 4B). In contrast, when printed with an SLA 3D printer, layers with symmetrical straight smooth margins appeared (Figure 4C).

In the present study, the differences in digital data deviation could be explained by the density of polygons formed by IOS and 3D printing data; Rapidform software was used for data analysis. IOS and 3DP data were selected and tooth data were investigated from three areas: teeth #13 (MxC), #14 (MxFPM), and #16 (MxFM) (Figure 5). Typically, IOS data showed a higher density (polygons per unit area) relative to 3DP data. When polygon images acquired by software were investigated, the stitch density of various types of triangular polygon shapes was high in complex shapes with many anatomical details. Triangle polygons overlapped by stitching between IOS groups showed a good match (Figure 5A,B,C2). Further, between 3D model data, triangular polygon density and stitched shapes were different according to the 3D printed model (Figure 5A,B,C3–5).

For in-depth analysis of the printing processes of three different types of 3D printed models, teeth #13 (MxC), #14 (MxFPM), and #16 (MxFM) were selected and investigated using FE-SEM images (Figure 6). In the DLP model, examination of the cavities in the #14 (MxFPM) area showed that the layers were insufficient, and that the flow of the hardened resin material was uneven. When layers were added in #13 (MxC), each layer stayed intact in a straight line (Figure 6A). In the FDM model, #14 (MxFPM) and #16 (MxFM) areas showed thick layers and the margins were not prominent, while the order of layers when layering filament material did not match or was uneven (Figure 6B). In the SLA model, the order of layers in the entire area was clean, the flow of resin was even, and the hardened resin material in between layers showed good adhesion (Figure 6C).

## 4. Discussion

The objective of the present study was to confirm the presence of a mutual relationships between IOS and 3D printing in digital workflow which could affect the accuracy of digital impressions. For this, the reference model was scanned using IOS and the accuracy of the 3D printed model was analyzed. Based on this, it can be assessed whether the compatibility between the two types of equipment could have a major influence on the clinical process. The null hypothesis was that there would be no differences in compatibility with respect to the accuracy values between various combinations of technologies, when the result accounting for both IOS and 3D printing processes (Ref-IOS/3DP) and results accounting for only 3D printing (IOS-3DP) were compared. Measurement results showed that the median trueness of each group varied, and that there was specific combination of IOSs and 3D printers that were compatible with each other. Therefore, the null hypothesis that there would be no difference in the accuracy between Ref-IOS/3DP and IOS-3DP was rejected.

Every year, new upgraded IOSs with their own unique operating principle and operational characteristics are introduced to the market and are becoming more commonplace in clinics based on efficiently improved performance [2,20]. In the present study, recently introduced CS3600, i500, and Trios3 IOSs were used to scan a phantom model, and the results were compared with reference data from scanning with a reference scanner to assess trueness (Ref-IOS). The accuracy of CS3600 was inferior to those of Trios3 and i500, while the most accurate IOS was found to be the i500. Such findings were consistent with the results of a study by Kim et al. regarding the comparison of trueness between various IOSs and a complete-arch scan of multiple implant cylinders. i500 and Trios3 showed excellent accuracies among the various IOSs [20]. The size of errors that appeared in the color-coded map in the present study was smaller than that of the previous study. There are several causes of IOS data error, and in particular, errors may occur during the process of aligning images of large and simple objects [21,24]. Scan accuracy and deviation have been reported to generalize inaccurate and false information due to error accumulation in the image recombination process or overlapping data stitching error [34]. When the polygon density of IOS scanned data was investigated in the present study, the CS3600 group showed higher polygon density than i500 and Trios 3 (Figure 5A1–C1), but it also showed poorer accuracy. The study by Kim et al. reported that the shape of polygons for IOS data could cause differences in accuracy according to the difference in the distribution of equilateral and skinny triangles. For example, the PlanScan group generated polygons with a high density, but its measured accuracy was inferior, which was consistent with the tendency found in the present study [2,35]. In the present study, the i500 group showed many equilateral triangles, whereas the CS3600 and Trios3 groups showed many skinny and equilateral triangles, along with some uneven lines and surfaces (Figure 5, per unit area A1–C1 of A2–C2 red box).

The present study performed intraoral scans on a quadrant-arch model, and when compared to studies that used complete-arch models, the median trueness values were relatively accurate. In an in vivo study by Ender et al. and an in vitro study by Renne et al., the quadrant-arch scan was more accurate than a complete-arch scan. Unscanned areas, which are represented as holes, are automatically patched by the software [26,27,36]. Ender et al. reported that the accuracy of scanned data may be affected by the algorithm of scan software and that deviation gradually increases as the error in the anterior teeth region accumulates and carries over to the distal end of the arch [27,37]. Despite this, Braian et al. reported that accuracy increases during the overlap process when scanning a complex-shaped object with a short span, and in particular, because stitching is easier when scanning an occlusal surface with complex geometry, there may be fewer errors than when scanning a smooth surface [2,20,21]. Figure 3A shows the assessment results of trueness of IOS data of a quadrant-arch from color map analysis in the present study. Park et al. analyzed the trueness of scanned inlay cavity data and expressed the results as a color map. When the maxillary first premolar and first molar were compared within the same range as the present study (−100 to +100 μm), the cervical area, especially the surrounding pattern in the box area of cavities showed less red color. Such a difference may appear because of a difference in accuracy according to the laboratory environment, surface characteristics of the reference model, scan strategy of the investigator, weight of the scanner, or wand size [3]. In the present study, anterior (incisor) and posterior (molar) regions showed the largest errors in the post-printing color map (Figure 3B and Figure 4C). It was confirmed that errors were more diverse according to the digital model region, as compared to reference IOS images, because the printed model contained a mixture of expanding and contracting areas.

When the 3D printed models based on an intraoral scan of the quadrant model were analyzed in the present study, trueness became much worse as compared that of the intraoral scan process alone. However, the trueness values in all groups were within the clinically allowable range [9]. In the measurement results that accounted for only the 3D printing process (IOS-3DP), there were differences in median trueness between the groups, with FDM showing a high value of 75.9 μm and DLP showing a low value of 49.5 μm, while SLA showed a median value of 61.2 μm. In the study by Kim et al. on a printed complete-arch model, fused filament fabrication showed a value of 241 μm and DLP showed a high value of 260 μm, whereas SLA showed a low trueness value of 237 μm [32]. The accuracy of 3D printers can be affected by various causes, such as arch size (complete-arch or quadrant-arch), the 3D printing process, and measurement method. Nestler et al., who explained the causes of deviations that occur during printing, compared photopolymerization-based and extrusion-based systems and reported that shrinkage and warpage were caused by incomplete polymerization of the resin monomer by light sources during printing. It was explained that DLP showed superior trueness compared with other printers because of better polymerization of resin [5,9,18]. Gjelvold et al. assessed deviations between the positions of surgical guides for computed tomography implant surgery fabricated by desktop 3D printers based on two different techniques (DLP and SLA). They reported that the SLA technique showed a slightly higher deviation in specific parts of the guide, as compared to the DLP technique, and presented the cause of the difference between the mean deviation of the dental implant position [18]. Other studies reported that one of the advantages of DLP and SLA printers was less shrinkage as compared to other 3D technologies, including FDM [16,31,38,39].

The present study used images to show the difference between the FDM technique (extrusion-based) that applies heat for printing and SLA and DLP techniques (photopolymerization-based) that harden light curable resin by laser or ultraviolet light. The fact that using a laser allowed for a soft and smooth finish and showed higher resolution was confirmed based on magnified electron microscopic findings (Figure 6), which was contrary to the FDM group that showed a pattern of a stepped layer. Meanwhile, Kim et al. compared images of three different models produced by three different 3D printing methods, and showed that models printed with SLA, FDM, and DLP 3D printers all appeared to have the same smooth surface [32]. In the present study, the shape of the support was presented by a high-resolution photograph for a clear comparison of differences in the surfaces of models produced by different printing methods. The inlay model printed by the FDM method did not show a clear crown margin or clear boundary lines, whereas SLA and DLP models showed clear marginal lines (Figure 4). This part is an essential element for excellent clinical outcomes. A study by Cole et al., which compared and assessed conventionally fabricated clear retainers and 3D printed retainers, reported that 3D printed retainers had a higher trueness value and that the size of the model generally changed during the 3D printing process; thus, accuracy may be lowered [17]. It is necessary for 3D printed dental models to have high dimensional accuracy, while the 3D printing method and specifications that affect surface resolution are also important. Accuracy is determined mostly by the characteristics of SLA, DLP, and FDM printers, but material-related geometry of layer thickness and 3D printing build orientation could also influence the warpage effect and the dimensional accuracy of shrinkage [9,10,31,32].

The present study conducted a comparative analysis of trueness values that accounted for only the 3D printing process (IOS-3DP) and for both scanning and printing processes (Ref-IOS/3DP). The results showed that there was a difference in accuracy according to the compatibility flow between IOS and 3D printing (Table 4). Based on this, the association between three different IOS groups and three different 3D printer groups could be assessed. When assessing the accuracy of models that were 3D printed based on orally scanned data, it was determined that different results may be found depending on different combinations because of the characteristics and advantages/disadvantages of each type of equipment and technology. The combination that showed the best result among IOS-3DP trueness with only 3D printing was the DLP printer and i500scanner, while the most suitable scanner for the FDM printer was the CS3600. On the other hand, it was confirmed that the SLA printer generally worked well with all scanners (Table 3). When assessing both IOS and 3D printing, the combination that showed the best result for Ref-IOS/3DP trueness was the i500 scanner and DLP printer. The FDM printer was suitable for use with the CS3600 scanner but had a poor mutual relationship with the i500 scanner (Table 3). Trios3 and the SLA printer were found to be a good combination. Trueness values found in the present study were all less than 43 to 78 μm, which would be sufficient for the purpose of assessing occlusion with a contact point for prostheses. Median trueness values of the 3D printed dental models were clinically acceptable according to the guidelines [9,32,40]. However, the results also showed that combining different scanners and printers could lead to different results.

The laboratory conditions were kept as accurate as possible and as close to the natural environment, but the study was not able to fully reproduce the conditions in actual clinical settings. As a result, there were some limitations. The experiment used a maxillary quadrant-arch typodont model made of Co-Cr metal for scanning; the study did not test dentin, enamel, or soft tissues that are typically encountered in actual clinical practice. Moreover, the present study was conducted under a constant relative humidity to minimize environmental bias, but it did not consider patient-related variables such as patients with limited mouth opening during intraoral scanning and interference by the cheek, tongue, saliva, and blood during scanning. Various combinations of IOSs and 3D printers were assessed in the present study and it was discovered that they have mutual compatibility. Therefore, when implementing equipment in the clinical setting, it is important to compare the various advantages and disadvantages of each equipment; however, compatibility should also be considered when choosing a system that suits each treatment environment. Finally, development of IOSs and 3D printer produce data that would benefit reduced costs, increased speed of manufacture, and more efficient, minimally invasive treatments for the patients. Accordingly, additional studies related to this are needed.

## 5. Conclusions

Based on the assessment of accuracy considering mutual compatibility when using a combination of IOSs and 3D printers within the limitations of the present study, the following conclusions were derived:

- In the Ref-IOS group, the i500 scanner was accurate (*p* < 0.05).
- In the Ref-IOS/3DP group, the best match was the combination of i500 and DLP, while Trios3 and i500 were more suitable for an SLA printer. Meanwhile, the FDM printer was suitable for use with the CS3600 (*p* < 0.05).
- In the IOS-3DP group, the best match was the combination of i500 and DLP, while CS3600 and FDM were suitable for use together. Meanwhile, the SLA printer worked well with all scanners (*p* < 0.05).
- The mutual relationship between IOS and the 3D printer varied depending on the combination. Although no equipment could be classified as being the most accurate, the DLP printer was most accurate in this study, regardless of which scanner it was combined with (*p* < 0.05).

## Figures and Tables

**Table 1:** Characteristics of scanners used in this study.

System	Manufacturer	Scanner Technology	Light Source	Acquisition Method	Necessary of Coating
Identica T500	MEDIT Corp.	Phase-shifting optical triangulation	Blue LED ^1^	Video	None
CS3600	Carestream Dental	Active triangulation (Stream projection)	Light	Video	None
i500	MEDIT Corp.	Dual camera optical triangulation	Light	Video	None
Trios3	3shape	Confocal microscopy	Light	Video	None

^1^ LED: light-emitting diode.

**Table 2:** Characteristics of 3D printers.

3D Printer	Category	Additive Manufacturing Process	Techniques	Layer Thickness	Print Time
Formlabs Form2	Vat photopolymerization	SLA technology that operates by heating a hard-photosensitive liquid resin into a hard-solid 3D form made of plastic through the application of a powerful laser beam with vat polymerization-based technology. Accordingly, after projecting a laser beam moved by micromirrors, the resin layer is cured to produce the product. SLA could produce complex shapes with high functional resolution and produce smooth and precise lines to produce good models.	Stereolithography apparatus (SLA)	100 μm	2 h 54 min
Veltz3D D2	Vat photopolymerization	DLP technology is similar to that of SLA, but it uses a high intensity light beam instead and the desired shape is formed as the liquid photopolymer resin hardens. However, rough lines may be produced, and pixels may be displayed depending on the resolution of the digital light projector. This means that the resolution of the projector is reflected in the quality of the final product. DLP has good accuracy and produce smooth surfaces.	Digital light processing (DLP)	100 μm	50 min
Flashforge Creator Pro	Material extrusion	One of the popular 3D printers that represents AM technology is the FDM printer with extrusion-based technology. A pressure-based process heats thermoplastic or composite filament material in the bottom layer to its melting point and releases it layer by layer to produce a pressure-assisted 3D printed product. The material used for the FDM is relatively inexpensive and can have a high mechanical strength.	Fused deposition modeling (FDM)	100 μm	3 h 12 min

**Table 3:** Comparison of median trueness values by all data capture principle (unit: μm).

Variable	N	Ref-IOS Data	Ref-IOS/DLP Data	Ref-IOS/FDM Data	Ref-IOS/SLA Data	IOS-DLP Data	IOS-FDM Data	IOS-SLA Data
		Median (Q1–Q3)						
CS3600	10	30.2 (27.1–34.9) a	59.5 (57.9–66.1) a	64.3 (63.8–68.1) b	57.1 (46.7–64.8) ab	51.8 (49.8–55.8) a	73.3 (71.8–75.1) b	61.5 (58.1–68.3) a
i500	10	23.5 (20.8–25.9) b	43.2 (38.5–46.1) b	81.9 (79.9–85.2) a	65.5 (62.8–68.9) a	46.2 (44.4–48.4) b	77.6 (74.9–79.9) a	60.2(56.6–63.1) a
Trios3	10	26.9 (24.5–28.6) ab	44.8 (39.2–48.2) b	78.8 (74.5–81.5) a	56.6 (47.9–60.5) b	50.8 (47.1–54.7) ab	78.8 (75.1–81.1) a	61.2(56.9–66.3) a
Total	30	26.6 (24.5–28.9)	47.1 (41.6–58.2)	76.3 (66.5–81.5)	59.8 (53.5–65.5)	49.5 (46.4–53.5)	75.9 (73.3–79.1)	61.2 (57.5–65.8)
df	-	2	2	2	2	2	2	2
Chi-square	-	15.4	19.6	19.1	8.7	10.5	11.5	0.7
*p* value	-	<0.001	<0.001	<0.001	0.013	0.005	0.03	0.695

df, degrees of freedom. Statistically significant at *p* < 0.05. Bonferroni: a>b. Median values used for statistical analysis. Different lowercase letters within the same column indicate statistical difference between Ref-IOS, Ref-IOS/3DP and IOS-3DP overall digital data capture principles. The “a” indicates higher value of each data while the “b” indicates lower value of each data. There is a significant difference between “a” and “b” while there is no significant difference between the same lowercase letters. If there is an “ab”, there is no significant difference between the “ab” and the “a”, and between the “ab” and “b” as well (multiple comparison by Mann-Whitney *U* test) (*p* < 0.05).

**Table 4:** Comparison of median trueness values of IOS-3DP and Ref-IOS/3DP deviations.

Variable	IOS-DLP―Ref-IOS/DLP	IOS-FDM―Ref-IOS/FDM	IOS-SLA―Ref-IOS/SLA
CS3600	51.8b―59.5a	73.3a―64.3b	61.5a―57.1b
i500	46.2a―43.2b	77.6b―81.9a	60.2b―65.5a
Trios3	50.8a―44.8b	78.8a―78.8a	61.2a―56.6b
Sum of Squares	3.408(143.092)	14.308(46.122)	87.363(191.065)
df	1(27)	1(27)	1(27)
Median square	3.408(5.300)	14.308(1.708)	87.363(7.076)
F	0.643(−)	8.376(−)	12.345(−)
*p* value	0.430(−)	0.007(−)	0.002(−)

Statistically significant at *p* < 0.05. Bonferroni: a>b. Repeated measures ANOVA group relations based on test pairwise comparisons are reported. The median trueness value used for statistical analysis showed no significant difference between Trios3-FDM and Ref-Trios3/FMD (*p* > 0.906) within the same column, and showed a significant difference between IOS-3DP and Ref-IOS/3DP (*p* < 0.001).
